# Machine learning in critical care: Improving prediction of mortality and intensive care unit stay after cardiac surgery

**DOI:** 10.1016/j.xjon.2026.101610

**Published:** 2026-01-31

**Authors:** Mihkel Meinberg, Zaeed Khan, Joel Jaskari, Erika Wilkman, Karl Lemström, Simo Särkkä, Simo Syrjälä

**Affiliations:** a Department of Anaesthesia and Intensive Care Medicine, Helsinki University Hospital and University of Helsinki, Helsinki, Finland; b Department of Electrical Engineering and Automation, Aalto University, Espoo, Finland; c Department of Computer Science, Aalto University, Espoo, Finland; d Department of Cardiac Surgery, Heart and Lung Centre, Helsinki University Hospital and University of Helsinki, Helsinki, Finland

**Keywords:** mortality risk, cardiac surgery, machine learning, risk assessment

## Abstract

**Objective:**

The European System for Cardiac Operation Risk Evaluation II (EuroSCORE II) is a multifactorial tool that assesses postoperative mortality risk after cardiac surgery but lacks several patient-specific and procedure-related factors. This study evaluated machine learning (ML) models against the EuroSCORE II for predicting in-hospital mortality and prolonged intensive care unit (ICU) stay after cardiac surgery.

**Methods:**

In this retrospective single-center study, data from 5606 adult patients undergoing primary open-heart surgery were analyzed. Six ML models were compared with EuroSCORE II using area under the receiver operating characteristic (AUROC) analysis for mortality and ICU length-of-stay predictions using a specified set of pre- and perioperative parameters. Feature importance for each prediction task was assessed using the random forest classifier.

**Results:**

Coronary artery bypass grafting was the most common procedure (50.2%), followed by aortic valve (20.7%), mitral valve (16.4%), thoracic aorta (8.8%), tricuspid valve (0.7%), and other procedures (3.1%). EuroSCORE II predicted a 2.26% mortality rate, whereas the actual in-hospital mortality was 1.5% (83 patients). The Gaussian Process (GP) classifier surpassed EuroSCORE II for prediction of in-hospital mortality with AUROC values of 0.877 versus 0.851 using preoperative data, reaching 0.886 when perioperative data were included. For prolonged ICU stay prediction, the GP classifier achieved AUROCs of 0.725 (preoperative data) and 0.749 (perioperative data).

**Conclusions:**

EuroSCORE II overestimated mortality despite a robust performance. Among tested ML models, the GP classifier demonstrated superior accuracy using preoperative data, further enhanced by perioperative parameters. Our results highlight ML's potential for improving risk assessment by its integration into next-generation cardiac surgery risk assessment tools.

**Figure:**
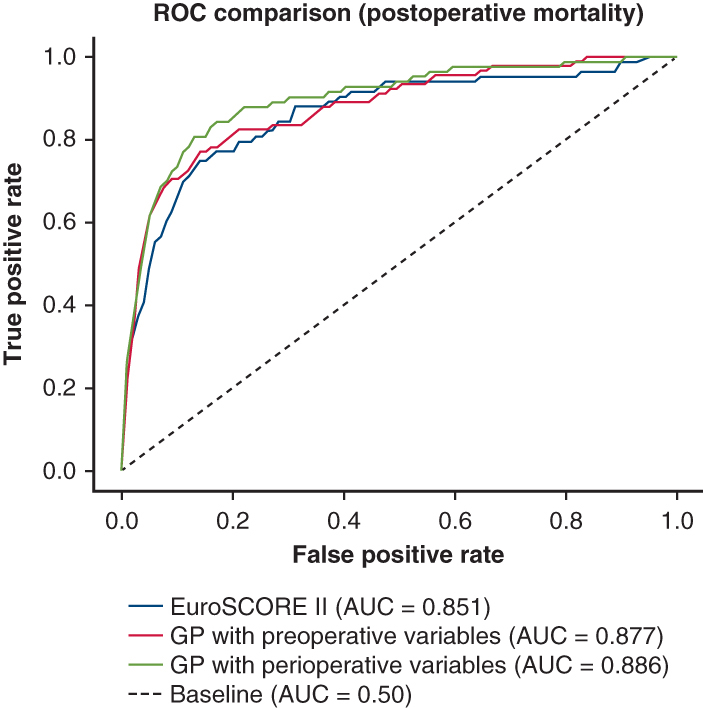
GP surpassed EuroSCORE II in prediction of postoperative mortality. *GP*, Gaussian process.

**Central Message**

ML models surpassed EuroSCORE II for postoperative mortality prediction and predicted extended ICU-LOS well using pre/perioperative data, with feature importance analysis identifying key factors.

**Perspective**

Compared with logistic regression-based algorithms, machine learning models offer improved processing of non-linear correlations in clinical settings, where multiple parameters are analyzed for outcome prediction. These findings further emphasize the growing value of machine learning models in cardiac surgery risk stratification and decision-making.

Open-heart surgery is used to treat certain cardiovascular conditions, the most common being bypass grafting in coronary artery disease and valve repair or replacement in valvular diseases. Early and late postoperative complications affect morbidity and mortality.^1^ The European System for Cardiac Operation Risk Evaluation II (EuroSCORE II) and the Society of Thoracic Surgeons (STS) score are widely used multifactorial risk calculators,^2^, ^3^, ^4^ although individual patient risks can be misestimated.^5^, ^6^, ^7^, ^8^, ^9^

Machine learning (ML) increasingly addresses medical classification tasks with encouraging results.^10^^,^^11^ ML classification algorithms include models with nonlinear classification decision boundaries, enabling more complex function fitting.^12^ Interest in ML methods for critical care predictive modeling has grown,^13^ and applications for predicting mortality and intensive care unit length of stay (ICU-LOS) are being developed.^14^, ^15^, ^16^

The study's aims were to (1) develop ML models using selected preoperative variables from adult patients undergoing primary open-heart surgery at a tertiary university hospital to predict postoperative mortality during index hospitalization (the primary end point), 30-day, and 90-day mortality, benchmarked against EuroSCORE II; (2) assess whether adding selected intraoperative variables (incision to ICU arrival) improved ML model performance; and (3) apply the ML approach to predict prolonged ICU-LOS (>27 hours postarrival).

## Patients and Methods

The institutional review board of the Helsinki University Hospital Ethical Committee approved this retrospective, single-center study (HUS/144/2020, approved February 17, 2020). Our unit administers a national secure data registry, which collects patient data for a research-dedicated data lake. Individual informed consent was waived because of the anonymous data management and the retrospective nature of the study. The study followed Declaration of Helsinki guidelines.

### Study Population

We searched our Cardiac Surgery Registry for patients who underwent primary open-heart surgery between August 1, 2014, and October 31, 2020, and identified 6844 patients. After we excluded 866 patients on the basis of the procedure type, we searched the institutional data lake for all available clinical information on 5978 patients, including surgical procedure data, perioperative surgical data, postoperative ICU data, hospital LOS, and survival. We selected only patients with complete datasets for ML model generation, yielding a final cohort of 5606 patients (Online Data Supplement 1).

All scheduled operations began after 8 am on weekdays and Saturdays during the study period. Additional second-round elective procedures may have followed the initial operations. Neither fast-track surgery nor Enhanced Recovery After Surgery protocols were used during the study period. After surgery, intubated and sedated patients were transferred to the ICU for postoperative care under standard operating procedures.

We chose EuroSCORE II as the reference on the basis of its validation in European populations, institutional adoption as the standard preoperative risk assessment tool, and data-field compatibility with our electronic health record system and surgery database. For mortality prediction analyses, we included preoperatively available variables on the basis of their assumed clinical significance and their inclusion in the EuroSCORE II risk calculator, as well as perioperative parameters to evaluate whether they improved ML model performance. Patient demographics, baseline characteristics, and analyzed variables are listed in Online Data Supplement 1.

Optimal use of surgical suites relies on postoperative ICU bed availability. Wagener and colleagues.^17^ found that patients with prolonged ICU stays comprised less than 10% of patients who underwent cardiothoracic surgery but consumed nearly one half of all ICU bed days. We therefore investigated whether ML models could predict prolonged ICU-LOS. With an uneventful postoperative course, patients are extubated within 6 to 12 hours, weaned off vasoactive medication, and transferred to the cardiac surgery ward or step-down unit within 24 hours of ICU arrival. Hemodynamic instability, inability to wean off from mechanical ventilation, rhythm issues, or neurologic problems are the main causes for extended ICU stay. Given that inappropriate ward bed occupancy and discharge delays can cause transfer delays from the ICU to the ward, we chose a 27-hour cut-off for predicting prolonged ICU-LOS.

### ML Methods

We studied 6 ML methods: (1) logistic regression, (2) random forest (RF), (3) Gaussian process classifier (GP), (4) multilayer perceptron neural network (NN), (5) support vector machine, and (6) gradient boosting. Training and model evaluation used nested stratified 5-fold cross-validation to preserve class distribution in each fold. Online Data Supplement 2 details model architectures, training procedures, regularization, performance evaluation, hyperparameter selection, and predictive accuracy assessment.

We evaluated the model's performance by the area under the receiver operating characteristic curve (AUROC), a common ML evaluation measure in medicine.^10^ We examined individual input feature importance using the RF classifier with the feature permutation method,^18^ which randomly permutes feature values across patients and calculates AUROC score decrease compared with uncorrupted data as a measure of feature importance.

Categorical variables are presented as counts and percentages and continuous variables as means or medians. Patient characteristics were compared between cohorts using Mann-Whitney *U* test, Student *t* test, Fisher exact test, and the χ^2^ test for independence. Baseline statistical analyses were performed using the IBM SPSS Statistical tool (version 29.0.0.0.(241); IBM Corp).

## Results

### Patient Characteristics

Among 5606 patients, observed in-hospital mortality was 1.5%, compared with EuroSCORE II-predicted mortality of 2.26%. Observed 30-day and 90-day mortality rates were 1.7% and 2.5%, respectively. A total of 1988 patients (35.5%) had ICU stays >27 hours (Online Data Supplement 1).

In univariate analysis, patients in the primary outcome group were significantly older, had a greater prevalence of moderate or severe pulmonary hypertension, critical preoperative state, poor or severely impaired left ventricular function, chronic lung disease, longer procedural times, more concomitant procedures, and lower hemoglobin levels in the first arterial blood gas analysis at ICU arrival. They were more likely to undergo emergency or salvage surgery, thoracic aortic surgery, and nighttime surgery. Their overall ICU-LOS was significantly longer. Patients with ICU-LOS >27 hours compared with ≤27 hours were older, had a greater prevalence of moderate or severe pulmonary hypertension, critical preoperative state, Canadian Cardiovascular Society class II-IV angina, and chronic lung disease. They were more often scheduled for emergency or salvage procedures, isolated coronary artery bypass grafting, thoracic aortic surgery, or aortic/mitral valve surgery with longer procedural times and more concomitant procedures (Online Data Supplement 1).

### ML Performance Evaluation

**Figure 1:**
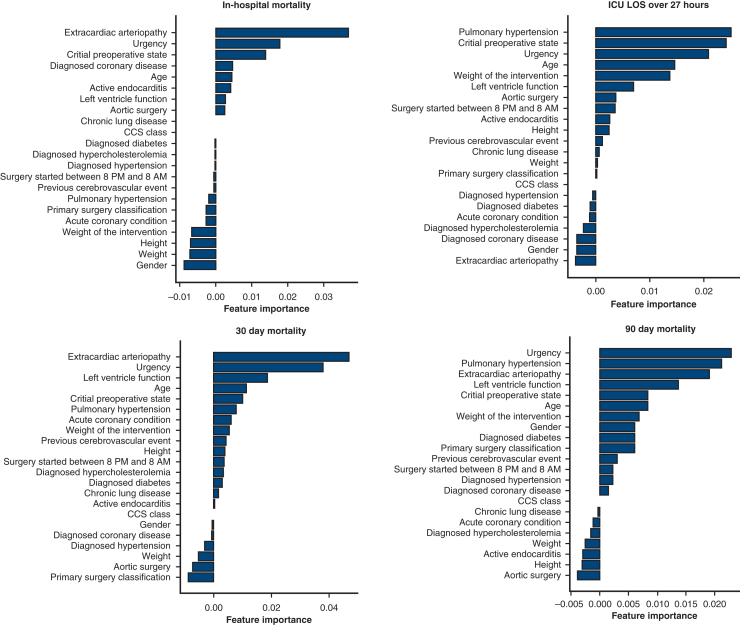
Feature importance of preoperative variables in each classification task, given by the random forest classifier. *CCS class*, Canadian Cardiovascular Society angina grade; *ICU-LOS*, intensive care unit length of stay.

**Figure 2:**
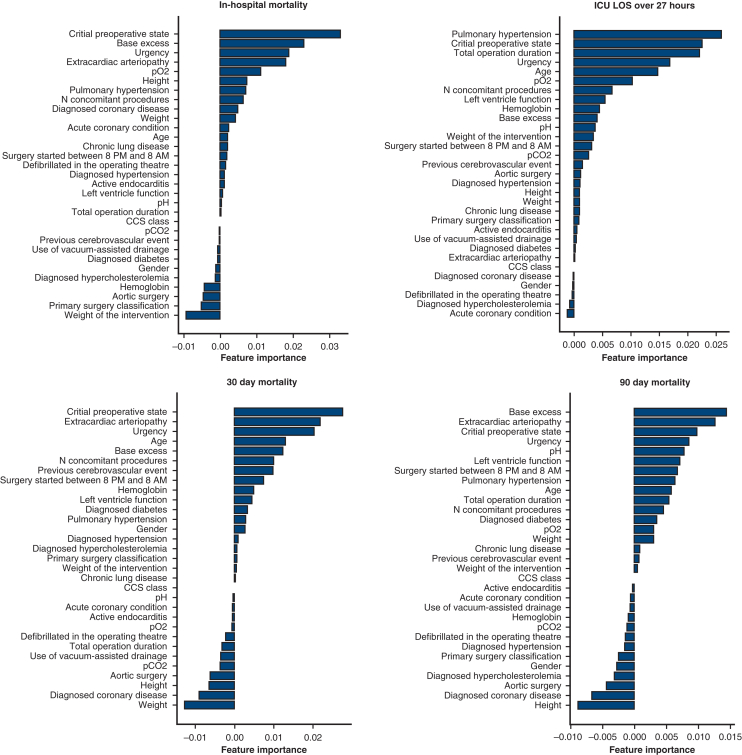
Feature importance of perioperative variables in each classification task, given by random forest classifier. *pO2*, Partial arterial pressure of oxygen; *CCS class*, Canadian Cardiovascular Society angina grade; *pCO2*, partial arterial pressure of carbon dioxide; *ICU-LOS*, intensive care unit length of stay.

EuroSCORE II and ML classifier performances based on preoperative data are presented in Table 1, with perioperative data in Table 2. The Brier scores for the ML classifiers with pre- and perioperative data are presented in Online Data Supplement 2 and Online Data Supplement 2 in Online Data Supplement 1. Feature importance in random forest classification tasks was analyzed with pre- and perioperative features ranked in Figures 1 and 2, respectively.

**Table 1:** Classification performance (AUROC) of the EuroSCORE II and ML classifiers with preoperative data only

Classifier	Primary end point	30-d mortality	90-d mortality	ICU LOS >27 h
EuroSCORE II (baseline)	0.851	0.821	0.813	0.708
LR, mean (SD)	0.861 (0.045)	0.853 (0.032)	0.814 (0.029)	0.724 (0.012)
NN, mean (SD)	0.584 (0.117)	0.593 (0.130)	0.581 (0.105)	0.716 (0.011)
RF, mean (SD)	0.860 (0.052)	0.845 (0.033)	0.818 (0.025)	0.719 (0.008)
GP, mean (SD)	0.877 (0.039)	0.857 (0.029)	0.823 (0.024)	0.725 (0.010)
SVM, mean (SD)	0.608 (0.076)	0.632 (0.063)	0.618 (0.056)	0.707 (0.013)
GB, mean (SD)	0.871 (0.041)	0.850 (0.039)	0.822 (0.038)	0.722 (0.008)

*ICU LOS*, Intensive care unit length of stay; *EuroSCORE II*, European System for Cardiac Operative Risk Evaluation II; *LR*, logistic regression; *SD*, standard deviation; *NN*, neural networks; *RF*, random forest; *GP*, Gaussian process; *SVM*, support vector machine; *GB*, gradient boosting.

**Table 2:** Classification performance (AUROC) of the ML classifiers with perioperative data

Classifier	Primary endpoint	30-d mortality	90-d mortality	ICU LOS >27 h
LR, mean (SD)	0.877 (0.044)	0.862 (0.032)	0.834 (0.020)	0.748 (0.015)
NN, mean (SD)	0.347 (0.055)	0.387 (0.051)	0.629 (0.145)	0.744 (0.012)
RF, mean (SD)	0.886 (0.044)	0.858 (0.032)	0.840 (0.029)	0.746 (0.011)
GP, mean (SD)	0.886 (0.039)	0.867 (0.034)	0.844 (0.023)	0.749 (0.017)
SVM, mean (SD)	0.771 (0.017)	0.769 (0.035)	0.746 (0.054)	0.743 (0.012)
GB, mean (SD)	0.889 (0.036)	0.853 (0.032)	0.847 (0.043)	0.749 (0.011)

*ICU LOS*, Intensive care unit length of stay; *LR*, logistic regression; *SD*, standard deviation; *NN*, neural networks; *RF*, random forest; *GP*, Gaussian process; *SVM*, support vector machine; *GB*, gradient boosting.

The GP classifier achieved the greatest AUROC scores across all tasks using preoperative data. All classifiers except the NN and support vector machine surpassed the EuroSCORE II baseline across all tasks. NN performed poorly in all tasks except prolonged ICU-LOS prediction. With perioperative data, the GP classifier surpassed all others in every prediction task. Similar to the results with the preoperative data, NN generally performed worst. The inclusion of perioperative data enhanced all classifiers' performance. On the basis of the Brier scores, the ML models are well calibrated when predicting the primary end point, 30- and 90-day mortality, but less well calibrated when predicting ICU-LOS. In the RF classifier analysis, critical preoperative state and procedure urgency contributed the most to the prediction of the primary outcome, regardless of the variable set. Among preoperative factors, extracardiac arteriopathy, diagnosed coronary disease, age, active endocarditis, left ventricle function, and aortic surgery were significant, whereas base excess, partial arterial pressure of oxygen, and the number of concomitant procedures improved the model's performance when perioperative factors were also considered. For prolonged ICU-LOS prediction, critical preoperative state, pulmonary hypertension, and urgency were key predictors. Age, the weight of the intervention, and left ventricular function contributed to preoperative model performance, whereas total operative duration, partial arterial pressure of oxygen, and the number of concomitant procedures improved perioperative model performance.

## Discussion

We investigated the performance of 6 ML models in predicting in-hospital, 30-day, and 90-day mortality in adult patients undergoing first-time cardiac surgery. We evaluated models separately with the selected preoperatively available variables (preoperative risk assessment) and additional intraoperative variables (postoperative risk update). We also used ML models to predict the prolonged ICU-LOS. Our results imply that ML models—especially GP in our cohort—offer the possibility to improve cardiac surgery risk assessment. Although the observed differences in AUROC values here are small in scope, more precise models combined with thorough risk-stratification strategies taking the clinical decision-making environment into account could offer clinical value.

Both EuroSCORE II and STS scores are widely used to assess the immediate in-hospital mortality risk after cardiac surgery. On the basis of logistic regression analyses, their patient-specific and procedure-related independent variables interact in a linear and additive fashion.^2^, ^3^, ^4^ This may hinder classification performance when comorbidities and physiological factors alter the interpretation of the remaining variables. For more complex interactions required for nonlinearly separable patterns, researchers must manually select features (eg, polynomial or bilinear) to improve the classification model.^19^ Conversely, ML models automatically learn complex nonlinear features and perform better with large annotated datasets.^12^^,^^20^^,^^21^ Open-source ML libraries enable fast implementation of the algorithms, accelerating modeling versus laborious manual feature engineering.

EuroSCORE II performs well in estimating operative mortality in all types of cardiac surgery (except for trauma, transplant, and ventricular assist device cases)^22^^,^^23^ but may overestimate the risk in isolated coronary artery bypass grafting,^24^ underestimate the risk in high-risk procedures,^25^ and perform somewhat-poorly in emergency procedures.^22^^,^^23^ We hypothesized that an insufficient set of risk factors and the logistic regression's conceptual basis—the model assumes variable independence and cannot learn nonlinear relationships—might explain these drawbacks. Nonetheless, collecting more patient-based, disease-based, and organizational-based data could improve the model's predictive power.^26^

ML methods can overcome the limitations of risk assessment based on preoperative variables, although perioperative data add decision-making complexity. Using ML models and EuroSCORE variables in smaller cohorts failed to improve mortality prediction versus logistic regression; however, larger cohorts showed modest prediction performance improvement of ML models.^27^^,^^28^ Even a slight improvement in discrimination of the ML model can lead to reassessment of fitness for surgery in a substantial number of patients. Similarly, in our study, the EuroSCORE II had reasonably high AUROC for in-hospital mortality, but most ML models performed better across outcome parameters.

Studies combining preoperative and intraoperative variables show improved short-term postoperative mortality prediction with ML modeling.^29^, ^30^, ^31^ This aligns with our findings, where the GP model incorporating pre- and intraoperative data showed superior performance. Prolonged ICU-LOS after cardiac surgery is linked to increased mortality, morbidity, and substantial ICU resource consumption.^32^^,^^33^ Absent universal ICU-LOS prolongation definitions result in varying and arbitrarily chosen cut-off criteria (24 hours to >10 days).^34^^,^^35^ The incidence of prolonged ICU-LOS ranges from 4% to 11%.^36^, ^37^, ^38^ At our institution, patients in the ICU with an uncomplicated postoperative course are transferred to the ward or step-down units the next day. Bed shortages in these units limit ICU bed availability for scheduled cardiac surgery, influencing the surgical suite performance. Optimal resource management is particularly pertinent for our unit, which holds national responsibility for thoracic transplantation, adult congenital heart disease surgery, and mechanical circulatory support treatment beyond elective and emergency cardiac surgery. Therefore, assessing prolonged ICU-LOS risk is crucial for efficient patient flow in hospitals offering cardiothoracic surgery services. Because our database design did not allow tracking certain types of postoperative complications prolonging ICU-LOS, ICU-LOS serves as a composite measure that reflects complications without requiring coding the individual complication.^39^ We chose the 27-hour prolonged ICU-LOS cut-off pragmatically, because patient transfers rarely occurred before noon but typically were completed by 4 pm. Although EuroSCORE II was not originally developed for prediction of ICU-LOS, we included its values as a reference to show that ML models can reasonably well predict prolonged ICU-LOS.

ML approaches are considered “black box” models with lower interpretability than logistic regression. Interpretability is central to model assessment, and feature importance analysis highlights variables most affecting the classification task. However, feature importance analysis addresses only the inclusion or exclusion of features in a model without information on how feature changes affect model performance. Feature importance analysis may enable extracting impactful factors from subpopulation analysis or guide multi-institutional data collection. Using SHapley Additive exPlanations, Sinha and colleagues^40^ identified the surgery type, age, creatinine clearance, and urgency class as the 4 most important variables affecting model performance in a large cardiac surgery cohort. Similarly, in our study, RF classifier assessments identified the critical preoperative state and the urgency of the intervention as key contributors to model performance. This underscores the importance of assessing locally relevant data because hospital and procedural differences can affect outcomes.

A limitation of the study is its retrospective design, introducing measurement and selection bias from excluding incomplete data. Single-center results should be cautiously extrapolated to other cardiothoracic surgery units. Several risk factors from intraoperative data (eg, transfusion of blood products, aortic crossclamp time, and cardiopulmonary bypass time) known to affect outcomes in cardiac surgical patients were not included. Although the STS score has demonstrated validity in North American populations, we used EuroSCORE II as the reference because of its validation in European populations, institutional workflow integration, and data infrastructure compatibility. We included only 6 ML models because of the computationally heavy hyperparameter selection period. Although our retrospective study demonstrated superior ML model performance, prospective validation is essential to evaluate model performance in live clinical environments before full deployment.

## Conclusions

ML models, especially GP, surpassed EuroSCORE II in predicting postoperative mortality. Despite GP's superior performance, its computational intensity should be taken into consideration when handling much larger datasets in the future. ML models successfully predicted prolonged ICU-LOS using preoperative or perioperative data; however, multicenter evaluation is needed to assess the generalizability of the proposed methodology. Finally, feature importance analysis proved useful for distinguishing essential factors from irrelevant inputs. Additional comparative insights may be obtained from future research that uses ML models and STS/EuroSCORE II scores. Our results, with other supportive studies, warrant ML-based model inclusion in developing future precise risk-assessment tools for patients who are scheduled to undergo cardiac surgery. Transferring our results in clinical application requires attention to implementation challenges, prospective validation, and procedural integration. Preoperative risk stratification followed by real-time perioperative support forms a roadmap that offers a clinical adoption pathway focused on measurable improvements in patient outcomes and resource use.

### Declaration of Generative AI and AI-Assisted Technologies in the Writing Process

During the preparation of this work the author(s) used Grammarly to improve readability of the manuscript, to check for grammatical errors and correct punctuation. After using this tool, the author(s) reviewed and edited the content as needed and take(s) full responsibility for the content of the publication.

## Conflict of Interest Statement

The authors reported no conflicts of interest.

The *Journal* policy requires editors and reviewers to disclose conflicts of interest and to decline handling or reviewing manuscripts for which they may have a conflict of interest. The editors and reviewers of this article have no conflicts of interest.
