# Supplementary material for: Machine learning in critical care: Improving prediction of mortality and intensive care unit stay after cardiac surgery
Source: JTCVS Open. 2026 Jan 31;30:101610. doi: 10.1016/j.xjon.2026.101610 (PMC13131176; doi:10.1016/j.xjon.2026.101610)
Supplement: Supplementary file 1 — Online Data Supplement 1 [file mmc1.docx]

Supplementary Material 1

Figure S1. Flowchart representation of the patient selection process

Table S1. Characteristics of the study population

Table S2. Abbreviations and acronyms

Figure S1. Flowchart representation of the patient selection process


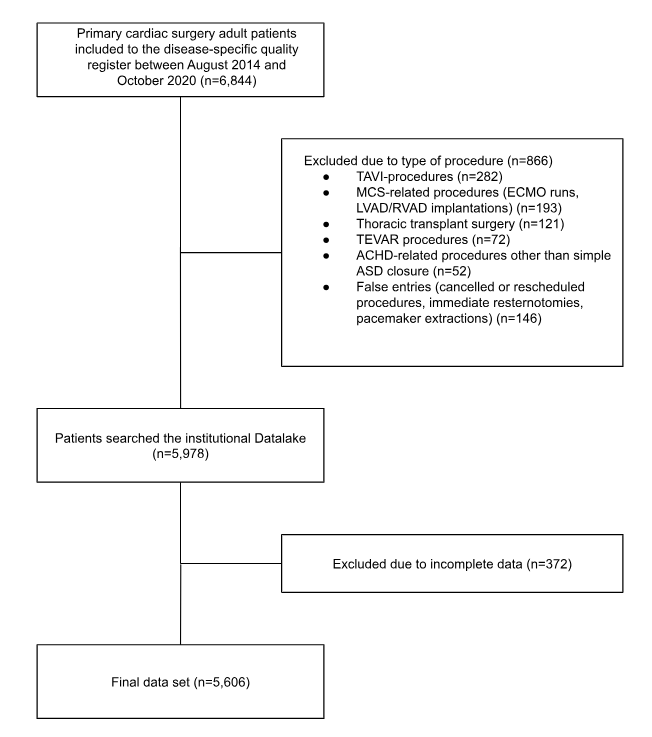


Table S1. Characteristics of the study population

| Characteristic | Study population (final dataset) | Died in hospital (primary endpoint) | Prolonged ICU stay (ICU LOS > 27 hours) |
| --- | --- | --- | --- |
| Patients, n (%) | 5606 (100.0) | 83 (1.5) | 1988 (35.5) |
| Female sex, n (%) | 1403 (25.0) | 23 (27.7) | 534 (26.9)* |
| Age (years), median (IQR) | 67 (59-75) | 72 (66-78)*** | 69 (62-77)*** |
| Pulmonary hypertension (PH), n (%) |  |  |  |
| Moderate PH | 1440 (25.7) | 37 (44.6)*** | 677 (34.1) *** |
| Severe PH | 443 (7.90) | 24 (28.9)*** | 293 (14.7) *** |
| Critical preoperative state, n (%) | 417 (7.44) | 44 (53.0)*** | 342 (17.2) *** |
| Active endocarditis, n (%) | 181 (3.2) | 6 (7.2) | 101 (5.1) *** |
| Left ventricle function, n (%) |  |  |  |
| Good (LVEF>50%) | 4171 (74.4) | 46 (55.4)*** | 1290 (64.9) *** |
| Moderate (LVEF 31-50%) | 1154 (20.6) | 15 (18.1) | 515 (25.9)*** |
| Poor (LVEF 21-30%) | 223 (4.0) | 12 (14.5)*** | 141 (7.1) *** |
| Very poor (LVEF ≤ 20%) | 58 (1.0) | 10 (12.0)*** | 42 (2.1) *** |
| Urgency class, n (%) |  |  |  |
| Elective | 3179 (56.7) | 18 (21.7)*** | 892 (44.9) *** |
| Urgent | 1782 (31.8) | 16 (19.3)** | 613 (30.8) |
| Emergency | 560 (10.0) | 33 (39.8)*** | 404 (20.3)*** |
| Salvage | 85 (1.5) | 16 (19.3)*** | 79 (4.0) *** |
| CCS class, n (%) |  |  |  |
| I | 781 (13,9) | 2 (2.4)** | 255 (12.8) |
| II | 974 (17.4) | 7 (8.4)* | 273 (13.7) *** |
| III | 1370 (24.4) | 16 (19.3) | 426 (21.4) *** |
| IV | 1068 (19.0) | 42 (50.6)*** | 519 (26.1) *** |
| Weight of operation, n (%) |  |  |  |
| Isolated CABG | 2669 (47.6) | 36 (43.4) | 803 (40.4) *** |
| Single non-CABG | 1530 (27.3) | 26 (31.3) | 533 (26.8) |
| Two procedures | 1060 (18.9) | 11 (13.3) | 465 (23.4) *** |
| Three or more procedures | 347 (6.2) | 10 (12.0) | 187 (9.4) *** |
| Thoracic aorta surgery, n (%) | 694 (12.4) | 15 (18.1)*** | 324 (16.3) *** |
| Chronic lung disease, n (%) | 482 (8.6) | 13 (15,6)*** | 226 (11.3) *** |
| History of cerebrovascular disease, n (%) | 86 (1.5) | 2 (2.4) | 59 (3.0) *** |
| Other variables |  |  |  |
| BMI (kg/m^2^), mean (SD) | 27.5 (4.83) | 27.53 (6.5) | 27.51 (5.1) |
| Preoperative potassium level (mmol/L),  median (IQR) | 4.1 (3.9-4.3) | 4.2 (4.0-4.5)** | 4.1 (3.9-4.3)*** |
| Preoperative sodium level (mmol/L),  median (IQR) | 139 (138-141) | 137 (135-140)*** | 139 (137-140)*** |
| Preoperative glucose level (mmol/L),  median (IQR) | 5.8 (5.4-6.6) | 6.7 (5.8-8.9)*** | 6.1 (5,5-7.3)*** |
| Night-time surgery  (Incision time between 8 PM and 8 AM,  n (%) | 112 (2.00) | 13 (15.7)*** | 100 (5.0) *** |
| Hypertension, n (%) | 772 (13.8) | 10 (12.0) | 300 (15.1)* |
| Dyslipidemia, n (%) | 390 (7.0) | 1 (1.2)* | 138 (6.9) |
| Diabetes mellitus, n (%) | 1074 (19.2) | 14 (16.9) | 407 (20.5) |
| Coronary artery disease, n (%) | 3333 (59.5) | 51 (61.4) | 1133 (57.0)** |
| Unstable coronary status (NSTEMI or unstable angina), n (%) | 2119 (37.8) | 41 (49.4)* | 772 (38.8) |
| Primary surgery classification, n (%) |  |  |  |
| CABG | 2816 (50.2) | 40 (48.2) | 886 (44.7) *** |
| Aortic valve surgery | 1163 (20.7) | 8 (9.6)** | 381 (19.2)* |
| Mitral valve surgery | 919 (16.4) | 12 (14.5) | 380 (19.1) *** |
| Tricuspid valve surgery | 42 (0.7) | 1 (1.2) | 20 (1.0) |
| Aortic surgery | 493 (8.8) | 16 (19.3)*** | 251 (12.6) *** |
| Other surgery | 173 (3.1) | 6 (7.2)* | 70 (3.5) |
| Intraoperative variables |  |  |  |
| Duration of the procedure (min),  median (IQR) ^a^ | 231 (186-277) | 262 (184-341) *** | 249 (195-301)*** |
| Use of vacuum-assisted drainage, n (%) | 314 (5.6) | 6 (7.2) | 119 (6.0) |
| Number of concomitant procedures, mean (SD) | 1.3 (0.6) | 1.7 (0.9)*** | 1.4 (0.7)*** |
| Epicardial defibrillation events, n (%)^b^ | 2411 (43.0) | 39 (47.0) | 877 (44.1) |
| Haemoglobin value in the first arterial blood gas analysis in the ICU (g/L), median (IQR)^c^ | 109 (100-118) | 101 (91-111)*** | 106 (98-115)*** |
| PaCO2 in the first arterial blood gas analysis in the ICU (kPa), median (IQR) ^c^ | 5.1 (4.7-5.6) | 5.2 (4.6-5.7) | 5.3 (4.8-5.8)*** |
| PaO2 in the first arterial blood gas analysis in the ICU (kPa), median (IQR) ^c^ | 22.9 (17-30.8) | 19.2 (11.1-32.7) | 19.3 (13.8-27.5)*** |
| pH of the first arterial blood gas analysis in the ICU (kPa), median (IQR) ^c^ | 7.39 (7.36-7.42) | 7.36 (7.31-7.41)** | 7.37 (7.33-7.41)*** |
| BE of the first arterial blood gas analysis in the ICU, median (IQR) ^c^ | -1.6(-2.8 - (-0.4)) | -3.1(-6.3 - (-1.1)*** | -2 (-3.6 - (-0.5))*** |
| EuroScore II, median (IQR) | 2.26 (0.4-4.1) | 18.23 (8.0-38.8)*** | 4.0 (1.9-10.8)*** |
| ICU LOS, h, median (IQR) | 23 (11-36) | 115 (38.0-269.0)*** | 70 (46-126)*** |

^a^, total time between incision and closure of the chest

^b^, the number of intraoperative epicardial defibrillation events

^c^, the laboratory value of the first postoperative arterial blood gas analysis taken upon arrival at the ICU

Abbreviations: *BE*, base excess, *BMI*, body mass index; *CABG*, coronary artery bypass graft surgery; *CCS,* Canadian Cardiovascular Society; *IQR,* interquartile range; *LVEF*, left ventricular ejection fraction; *NSTEMI,* non-ST-segment elevation myocardial infarction; *PaCO _2_*, partial pressure of arterial carbon dioxide; *PaO_2_*, partial pressure of arterial oxygen; *PH*, pulmonary hypertension; *SD,* standard deviation.

P-values: * = P < .05, ** = P < .01, *** = P < .001

Table S2. Abbreviations and acronyms

| Abbreviation/ acronym | Definition |
| --- | --- |
| ACHD | Adult congenital heart disease |
| ASD | Atrial septal defect |
| AUROC | Area under the receiver operating characteristic curve |
| BE | Base excess |
| BMI | Body mass index |
| CABG | Coronary artery bypass graft |
| CCS | Canadian Cardiovascular Society |
| ECMO | Extracorporeal membrane oxygenation |
| EuroSCORE II | European System for Cardiac Operation Risk Evaluation II score |
| ERAS | Enhanced Recovery After Surgery |
| GB | Gradient boosting |
| GP | Gaussian processes |
| ICU LOS | Length of stay in intensive care unit |
| ICU | Intensive care unit |
| LR | Logistic regression |
| LVAD | Left ventricular assist device |
| LVEF | Left ventricle ejection fraction |
| MCS | Mechanical circulatory support |
| ML | Machine learning |
| NN | Neural networks |
| NSTEMI | non-ST segment elevation myocardial infarction |
| PaCO_2_ | Partial arterial pressure of carbon dioxide |
| PaO_2_ | Partial arterial pressure of oxygen |
| PH | Pulmonary hypertension |
| RF | Random Forest |
| ROC | Receiver operating characteristic |
| RVAD | Right ventricular assist device |
| SD | Standard deviation |
| SHAP | Shapley Additive exPlanations |
| STS score | Society of Thoracic Surgeons score |
| SVM | Support vector machine |
| TAVI | Transarterial valve implantation |
| TEVAR | Thoracic endovascular aortic repair |
