# Supplementary material for: Machine learning in critical care: Improving prediction of mortality and intensive care unit stay after cardiac surgery
Source: JTCVS Open. 2026 Jan 31;30:101610. doi: 10.1016/j.xjon.2026.101610 (PMC13131176; doi:10.1016/j.xjon.2026.101610)
Supplement: Supplementary file 2 — Online Data Supplement 2 [file mmc2.docx]

Supplementary Material 2. Models, construction of the models, performance evaluation, optimal selection of hyperparameters and evaluation the predictive accuracy of the models.

Logistic regression (LR) estimates the relationship between one or more predictor variables and binary outcome variables^1,2^. In LR, a linear model and a logistic sigmoid function are combined to model the posterior probabilities of each class, and the sum of the probabilities of each class must be one^3^. Owing to the nonlinearity of the logistic sigmoid, no closed-form solution exists; therefore, iterative optimization methods, such as Newton–Raphson and gradient descent, must be applied^4^.

In a random forest (RF), several decision trees are constructed^5^. The decision trees are used as base learners, and their construction is based on Breiman’s bagging idea^6^. In a classification task, each decision tree casts a unit vote for the most popular class at the given input x^7^, and the final classification outcome is the class that obtained the majority vote^3^. RFs are robust to noisy data and overfitting^6^.

Gaussian processes (GP) are Bayesian nonparametric methods that can be applied in regression and classification tasks^8^. A mean function and a covariance function define the GP^9^. The basic principle of Gaussian process classification is to put GP priors over latent functions f (x) at the input x and squash them through a logistic function to obtain priors on class probabilities^10^. Integrals associated with GP classification are analytically intractable; therefore, analytic approximation methods, such as the Laplace approximation and expectation propagation, are needed for the integrals^8^.

Neural networks (NN) are nonlinear statistical machine learning methods that can be used in regression and classification tasks^3^. In a fully connected NN, linear operations and nonlinear activation functions are cascaded, with the linear operations being parameterized by a weight matrix and an intercept term. Features can be extracted from the inputs and applied for target modeling^3^. Like in logistic regression, the parameters of an NN are adjusted in the training phase with gradient descent-based methods to ensure that the model fits the training data.

Support vector machine is one of the classical machine learning models and it is based on four fundamental concepts^11^ : 1) the separating hyperplane, 2) the maximum-margin hyperplane, 3) the soft margin and 4) the kernel function. The separating hyperplane is a decision boundary that divides classes in feature space. The maximum-margin hyperplane is the separating hyperplane that maximizes the margin: the distance between the hyperplane and the nearest points from both classes. The soft margin, also known as the regularization parameter, specifies a trade-off between hyperplane misclassification and the size of the margin. Kernel function facilitates the SVM to find nonlinear decision boundaries, especially when the data is not linearly separable.

Gradient boosting is a boosting-based method that is applied to machine learning problems^12^ . The objective of gradient boosting is to approximate the function that maps inputs to their outputs by minimizing the expected loss. The approximation is constructed iteratively, using decision trees or other weak learners.

There are several so-called hyperparameters associated with the models not optimized directly by the learning algorithm but manually selected or optimized by a search algorithm. We used grid search to find the optimal hyperparameters and implemented all the models with scikit-learn^13^, thus concentrating on the hyperparameters available within the software package. For the LR classifier, the hyperparameters were the parameter penalty functions, specifically the L1-norm, L2-norm, or none. For the RF, the hyperparameters were the number of trees (100--500), the maximum depth of each tree (1--5), the minimum number of samples for each internal split (2--5), and the minimum number of samples for each leaf (1--5). For the GP classifier, we considered only the squared exponential, i.e., the radial basis function or kernel, as we observed computational difficulties with other kernels with the scikit-learn package. For the NN, the hyperparameters were the number of layers (1--3), the number of neurons in each layer (10--500), and the batch size (1--50).

Performance evaluation and hyperparameter selection

We performed a fivefold k-fold cross-validation strategy to evaluate the models' performance. In our k-fold cross-validation, the data are split into five nonoverlapping folds, and then we iterate over the folds by keeping one of the folds as hold-out test data while treating the rest of the data as the training set. Main results are reported as the average across the five folds. To optimize the hyperparameters, we conducted a nested stratified fivefold cross-validation process, where for each training set of the original k-fold process, we performed an additional fivefold cross-validation. We used the “inner” k-fold analysis results to determine the optimal hyperparameters for each task separately. The optimal hyperparameters found with the grid search are described in Table S2.1 and Table S2.2.

We also evaluated the predictive accuracy of the models using the Brier score^14^, a popular scoring metric in medical research (Table S2.3 and Table 2.4). The Brier score measures how well probabilities are calibrated to actual outcomes (accuracy of probabilistic predictions) while also considering the sharpness of those predictions^15^.

Tables

Table S2.1. Hyperparameters for preoperative models

| Classifier \ Task | In-hospital mortality | 30-day  mortality | 90-day  mortality | ICU LOS > 27 hours |
| --- | --- | --- | --- | --- |
| Logistic  Regression | Penalty: L1-norm | Penalty:  L1-norm | Penalty:  L1-norm | Penalty:  L1-norm |
| Random  Forest | Trees: 500  Max Depth: 5  Min samples split: 2  Min samples leaf: 4 | Tees: 500  Max Depth: 5  Min samples split: 2  Min samples leaf: 5 | Trees: 500  Max Depth: 5  Min samples split: 2  Min samples leaf: 2 | Trees: 500  Max Depth: 5  Min samples split: 5  Min samples  leaf: 1 |
| Neural  Network | Hidden layers: 1  Neurons per layer: 500,  Batch size: 10 | Hidden layers:  3  Neurons per layer: 500,  Batch size: 10 | Hidden layers:  1  Neurons per layer: 500,  Batch size: 10 | Hidden layers:  1  Neurons per layer: 200,  Batch size: 50 |
| Gradient Boosting | Trees: 100,  Max Depth: 1,  Min samples split: 2,  Min samples leaf: 2 | Trees: 100,  Max Depth: 1,  Min samples split: 2,  Min samples leaf: 5 | Trees: 100,  Max Depth: 1,  Min samples split: 2,  Min samples leaf: 5 | Trees: 100,  Max Depth: 2,  Min samples split: 2,  Min samples leaf: 4 |
| SVM | Reg. Parameter: 1.0,  Kernel: RBF | Reg. Parameter: 0.1,  Kernel: RBF | Reg. Parameter: 0.1,  Kernel: RBF | Reg. Parameter: 0.1,  Kernel: RBF, |

Table S2.2 Hyperparameters for perioperative models

| Classifier \ Task | In-hospital mortality | 30-day  mortality | 90-day  mortality | ICU LOS > 27 hours |
| --- | --- | --- | --- | --- |
| Logistic Regression | Penalty: L1-norm | Penalty: L1-norm | Penalty: L1-norm | Penalty: L1-norm |
| Random Forest | Trees: 500  Max Depth: 5  Min samples split: 2  Min samples leaf: 5 | Tees: 500  Max Depth: 4  Min samples split: 2  Min samples leaf: 5 | Trees: 500  Max Depth: 5  Min samples split: 2  Min samples leaf: 4 | Trees: 500  Max Depth: 5  Min samples split: 2  Min samples leaf: 1 |
| Neural Network | Hidden layers: 2  Neurons per layer: 500,  Batch size: 10 | Hidden layers: 2  Neurons per layer: 500,  Batch size: 10 | Hidden layers: 1  Neurons per layer: 200,  Batch size: 1 | Hidden layers: 1  Neurons per layer: 500,  Batch size: 10 |
| Gradient Boosting | Trees: 100,  Max Depth: 1,  Min samples split: 2,  Min samples leaf: 5 | Trees: 100,  Max Depth: 1,  Min samples split: 2,  Min samples leaf: 1 | Trees: 500,  Max Depth: 1,  Min samples split: 2,  Min samples leaf: 4 | Trees: 100,  Max Depth: 2,  Min samples split: 2,  Min samples leaf: 3 |
| SVM | Reg. Parameter: 0.1,  Kernel: RBF | Reg. Parameter: 1.0,  Kernel: RBF | Reg. Parameter: 1.0,  Kernel: RBF | Reg. Parameter: 0.1,  Kernel: RBF, |

Table S2.3. Predictive accuracy (Brier scores) of ML classifiers with preoperative data only

| Classifier | Primary endpoint | 30-day mortality | 90-day mortality | ICU LOS > 27 hours |
| --- | --- | --- | --- | --- |
| LR | 0.0145 | 0.0174 | 0.0242 | 0.1874 |
| NN | 0.0302 | 0.0301 | 0.0377 | 0.1900 |
| RF | 0.0148 | 0.0174 | 0.0241 | 0.1930 |
| GP | 0.0144 | 0.0172 | 0.0238 | 0.1872 |
| SVM | 0.0156 | 0.0186 | 0.0253 | 0.1934 |
| GB | 0.0145 | 0.0173 | 0.0240 | 0.1880 |

Table S2.4. Predictive accuracy (Brier scores) of ML classifiers with perioperative data

| Classifier | Primary endpoint | 30-day mortality | 90-day mortality | ICU LOS > 27 hours |
| --- | --- | --- | --- | --- |
| LR | 0.0131 | 0.0156 | 0.0223 | 0.1808 |
| NN | 0.0366 | 0.0376 | 0.0388 | 0.1834 |
| RF | 0.0136 | 0.0161 | 0.0226 | 0.1881 |
| GP | 0.0132 | 0.0158 | 0.0223 | 0.1796 |
| SVM | 0.0145 | 0.0171 | 0.0238 | 0.1848 |
| GB | 0.0134 | 0.0162 | 0.0226 | 0.1802 |

1. Ernest YB, Daniel AA. A Review of the Logistic Regression Model with Emphasis on Medical Research. *Journal of Data Analysis and Information Processing*. 2019;07(04):190-207. doi:10.4236/jdaip.2019.74012

2. Schober P, Vetter TR. Logistic Regression in Medical Research. *Anesth Analg*. 2021;132(2):365-366. doi:10.1213/ane.0000000000005247

3. Hastie T, Tibshirani R, Friedman J. The Elements of Statistical Learning, Data Mining, Inference, and Prediction. *Springer Series in Statistics*. Published online 2009. doi:10.1007/978-0-387-84858-7

4. Jaskari J, Myllärinen J, Leskinen M, et al. Machine Learning Methods for Neonatal Mortality and Morbidity Classification. *IEEE Access*. 2020;8:123347-123358. doi:10.1109/access.2020.3006710

5. Fan Y, Dong J, Wu Y, et al. Development of machine learning models for mortality risk prediction after cardiac surgery. *Cardiovasc Diagn Ther*. 2022;0(0):0. doi:10.21037/cdt-21-648

6. Cutler A, Cutler DR, Stevens JR. Ensemble Machine Learning. Published online 2012:157-175. doi:10.1007/978-1-4419-9326-7_5

7. Kulkarni VY, Sinha DPK. Pruning of Random Forest Classifiers: A Survey and Future Directions. *2012 International Conference on Data Science & Engineering (ICDSE)*. 2012;1:64-68. doi:10.1109/icdse.2012.6282329

8. Rasmussen CE, Williams CKI. *Gaussian Processes for Machine Learning*. Vol 1. The MIT Press; 2005. doi:10.7551/mitpress/3206.001.0001

9. Nickisch H, Rasmussen CE. Approximations for Binary Gaussian Process Classification. *Journal of Machine Learning Research*. 2008;9:2035—2078. http://jmlr.org/papers/v9/nickisch08a.html

10. Urtasun R, Darrell T. Discriminative Gaussian process latent variable model for classification. *Proceedings of the 24th international conference on Machine learning*. Published online 2007:927-934. doi:10.1145/1273496.1273613

11. Noble WS. *What Is a Support Vector Machine?* Vol 24.; 2006. http://www.nature.com/naturebiotechnology

12. Bentéjac C, Csörgő A, Martínez-Muñoz G. A comparative analysis of gradient boosting algorithms. *Artif Intell Rev*. 2021;54(3):1937-1967. doi:10.1007/s10462-020-09896-5

13. Pedregosa F, Varoquaux G, Gramfort A, et al. Scikit-learn: Machine Learning in Python. *Journal of Machine Learning Research*. 2011;12:2825—2830. http://jmlr.org/papers/v12/pedregosa11a.html

14. Redelmeier DA, Bloch DA, Hickam DH. Assessing predictive accuracy: How to compare brier scores. *J Clin Epidemiol*. 1991;44(11):1141-1146. doi:10.1016/0895-4356(91)90146-z

15. Rufibach K. Use of Brier score to assess binary predictions. *J Clin Epidemiol*. 2010;63(8):938-939. doi:10.1016/j.jclinepi.2009.11.009
